# Drain site enterocutaneous fistula after hyperthermic intraperitoneal chemotherapy

**DOI:** 10.1002/jgh3.12446

**Published:** 2020-10-28

**Authors:** Navin Kumar, Kanak Lata, Mukur Dipi Ray

**Affiliations:** ^1^ Department of surgical oncology All India Institute of Medical Sciences New Delhi India; ^2^ Department of nuclear medicine All India Institute of Medical Sciences New Delhi India

**Keywords:** cytoreductive surgery, enterocutaneous fistula, hyperthermic, intraperitoneal chemotherapy

## Abstract

Spontaneous sigmoid colon perforation after cytoreduction surgery and hyperthermic intraperitoneal chemotherapy (HIPEC) is a rare complication. It is more commonly seen with mitomycin‐based HIPEC. This case study's patient presented with pus discharge at the drain site after 4 weeks of surgery. The symptoms persisted after conservative treatment. High suspicion after the feculent smell of the discharge fluidled to the prompt diagnosis of enterocutaneous fistula. There was limitedperforation with abscess formation, followed by fistula formation. The patient was treated successfully with surgery.

## Graphical abstract

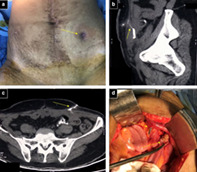

This case study's patient presented with pus discharge at the drain site after a few weeks of cytoreduction surgery and hyperthermic intraperitoneal chemotherapy (HIPEC). It was a limited perforation with abscess formation, followed by fistula formation. The patient was treated with a prompt surgical approach. Bowel edema with bacterial translocation may be the etiology of such complications.

## Introduction

A fistula is defined as an abnormal communication between two epithelialized hollow spaces or organs. Enterocutaneous fistula connects the bowel to the skin. It can be classified according to the source, output volume, and etiology. Fistulas from different organs have different types of outputs. Electrolyte and nutritional losses can vary accordingly. The low‐output, moderate‐output, and high‐output fistulas were defined as having drain volumes less than 200 mL/day, between 200 and 500 mL/day, and more than 500 mL/day, respectively. High‐output fistulas do not heal spontaneously, and these patients are at a higher risk of metabolic disturbances, fluid loss, and malnutrition. The prevalence of enterocutaneous fistulas in the general population is not known. However, postoperative prevalence of enterocutaneous fistulas is 1.5% for trauma,^1^ 3.6% for general surgery,^2^ and 15–35% for patients with Crohn's disease .^3^ The latter is the most common cause of spontaneous fistulas. These are classically treated with immunosuppressive drugs like thiopurines, biologic agents like tumor necrosis factor inhibitors, and antibiotics like metronidazole to reduce bowel inflammation and diarrhea.

Spontaneous fistula is a known but rare complication of cytoreductive surgery (CRS) and hyperthermic intraperitoneal chemotherapy (HIPEC). This will usually result in fecal peritonitis but occasionally results in a fistula as outlined in this report. The occurrence of such complications depends on the extent of the peritoneal disease. Mortality ranges from 36 to 44% in the literature.^4^ In 15% of cases, the site of perforation in fecal peritonitis remains unclear.^5^ The peritoneal trauma due to the direct toxic effect of HIPEC with extensive cytoreduction surgery, occasionally combined with the severe chemotherapy‐induced neutropenia, may be the reason why.^6^ This study aims to report a case of spontaneous limited perforation with abscess formation followed by enterocutaneous fistula formation in an ovarian carcinoma patient after CRS with HIPEC in our tertiary care center.

## Case report

**Figure 1:**
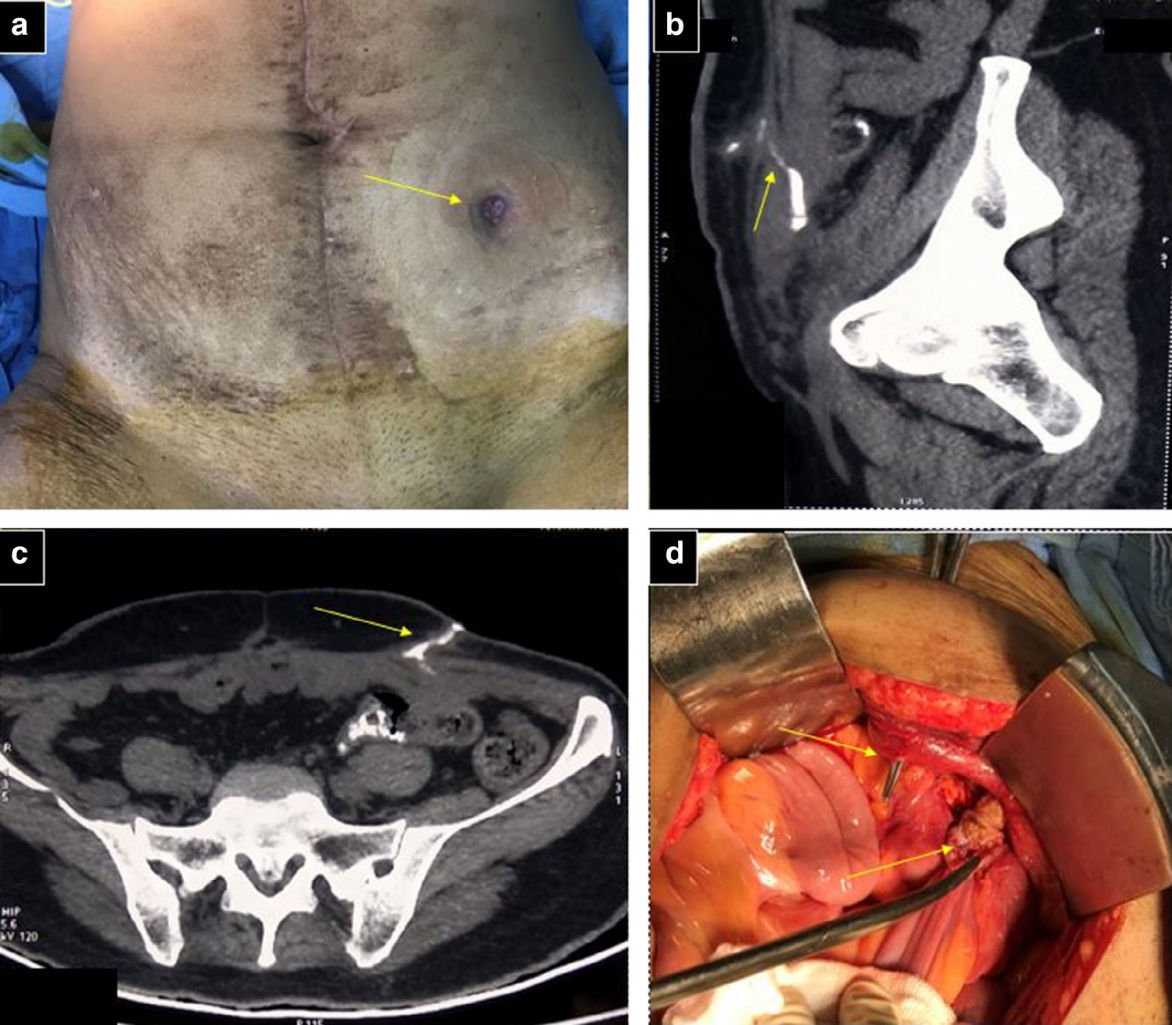
(a) Fistulous tract opening in left iliac fossa (yellow arrow). Computed tomography fistulogram shows thick‐walled communicating tract from the sigmoid colon to exterior (yellow arrow) in sagittal (b) and axial (c) views. (d) The intraoperative demonstration of enterocutaneous fistula (yellow arrow).

A 48‐year‐old female was evaluated outside of our center and diagnosed with left ovarian carcinoma. She underwent total laparoscopic hysterectomy with bilateral salpingo‐ophrectomy and received seven cycles of taxane/platinum (TP regimen)‐based adjuvant chemotherapy. After approximately 18 months of disease‐free interval, she was evaluated and diagnosed with recurrent ovarian carcinoma, stage IIIC. In a multidisciplinary tumor board discussion, she was planned for chemotherapy followed by surgical reassessment. After eight cycles of TP‐based chemotherapy, she was reevaluated with positron emission tomography‐computed tomography (PET‐CT). It showed multiple metabolically avid lesions at the surgical bed, omentum, right paracolic gutter, parietal wall, and suprapubic region, suggestive of partially responsive operable disease. Secondary cytoreduction surgery (total omentectomy and disease‐specific peritonectomy) with bilateral pelvic node dissection was performed. Intraoperatively, large omental caking was observed, 10 × 8 cm in size, with multiple peritoneal deposits in the bilateral paracolic gutter, right subdiaphragmatic region, pelvis, lesser omentum, and over segment 6 of the liver. The peritoneal carcinoma index was 13. Staged HIPEC was performed on postoperative day (POD) 1 because of intraoperative instability during the previous definitive surgery. The drug Cisplatin was used in 2 liters of normal saline perfusate at 41–43°C for 1 h. She recovered uneventfully. Histopathological features included high‐grade serous carcinoma and immunopositivity for WT1 and CK7. All retrieved pelvic nodes were free of malignancy. She developed a surgical site infection at the drain site in the left iliac fossa after 1 month of surgery, which worsened with conservative treatment. Later, she presented with fecal discharging sinus (Fig. 1a). Contrast‐enhanced CT with CT‐fistulogram revealed enterocutaneous fistula (Fig. 1b,c). Exploratory laparotomy with partial sigmoid colectomy and fistulous tract excision, followed by colocolic anastomosis, were performed. Intraoperatively, a fistulous tract from the sigmoid colon to left iliac fossa skin was identified (Fig. 1d). Postoperative recovery was uneventful.

## Discussion

Although enterocutaneous fistulas are a known complication of major surgery for ovarian cancer,^7^, ^8^ the delayed emergence of a fistula 4 weeks after surgery and CRS/HIPREC appears to be rare. The physiopathology of this postoperative complication is probably due to intraoperative/postoperative peritoneal bacterial contamination. Chua *et al*. showed that the morbidity and mortality of CRS/HIPEC were similar to those of other major gastrointestinal operations.^9^ These fistulas are significantly associated with the operative time, previous systemic chemotherapy or radiotherapy, the number of anastomoses, and the nutritional status. Kusamura *et al*. found that the extent of cytoreduction surgery and a dose of CDDP ≥240 mg were independent risk factors for digestive fistulas.^10^

Intestinal wall edema after CRS/HIPEC causes loosening of the intercellular tight junctions, and this helps in bacterial translocation.^11^ HIPEC aggravates visceral edema. Changes in gut microbiota and impaired immunity due to major surgeries are other probable factors for digestive fistulas. Nevertheless, these hypotheses are not proven, but a possible explanation could be an extensive systemic inflammatory response leading to the suppression of cell‐mediated immunity.^12^ In our tertiary care center, complete CRS/HIPEC is a complex and aggressive surgical procedure because of the heavy disease burden in the patients. Cisplatin is the most commonly used chemotherapeutic agent in HIPEC for ovarian malignancies. The duration of HIPEC was 60 min in this case. Staged HIPEC was used in this patient as her vitals during the index surgery (complete CRS) were unstable, preventing the administration of HIPEC in the same sitting. Duration of CRS was approximately 8.5 h. This major surgical trauma followed by HIPEC on postoperative day 1 could have caused the bowel edema and exacerbated the impaired immunity in the patient. In addition, the gut microbiota could be changed due to perioperative interventions of CRS/HIPEC, like preoperative mechanical bowel preparation, perioperative antibiotics administration, and the prolonged postoperative ileus‐decreasing intestinal clearance.

This patient presented with symptoms of surgical site infection at the drain site after 1 month of surgery. We advised medical treatment, but the patient did not respond to it and presented with fecal discharge from the same site. Evaluation with radiological imaging confirmed the diagnosis of enterocutaneous fistula, and the patient was treated curatively with surgical intervention. The principles of surgical management are to eliminate the source of infection, to reduce bacterial contamination with peritoneal lavage, and to prevent persistent or recurrent intraperitoneal infection with intraoperative drain placement.^13^ Broad‐spectrum antibiotics are also started for the coverage of the most common pathogens until the bacterial culture/sensitivity reports from intraoperative samples are obtained.

The role of the somatostatin analog octreotide for the treatment of digestive fistula remains controversial. It can significantly decrease the fistula output and hence reduces the time to fistula closure.^14^

Total parenteral/enteral nutrition helps in infection control and fistulous tract maturation. Fluid and electrolyte correction with abdominal sepsis control are key factors for normal intestinal function and motility.

The mortality reported in various series from postoperative peritonitis due to digestive fistulas is 10–47%.^13^, ^14^ However, this figure is reduced to 8% after CRS/HIPEC in our center (unpublished data from prospectively maintained departmental database). The reasons may be prompt surgical treatment delivery offered by the experts with a combined multidisciplinary approach.

In conclusion, postoperative enterocutaneous fistula after CRS/HIPEC is a rare entity, which can occur in patients treated with aggressive surgeries for high peritoneal carcinoma index. It may be due to bacterial translocation as a result of bowel edema after the surgery and intraperitoneal chemotherapy. Treatment principles do not differ from other forms of peritonitis. Resuscitation, control of sepsis, and nutritional support are the initial treatment modalities. Definitive surgery to restore gastrointestinal tract continuity, such as in our case, is warranted if the conservative approaches fail. A prompt multidisciplinary approach decreases morbidity and mortality.
